# A phase I study of pemetrexed (LY231514) supplemented with folate and vitamin B_12_ in Japanese patients with solid tumours

**DOI:** 10.1038/sj.bjc.6603321

**Published:** 2006-08-29

**Authors:** K Nakagawa, S Kudoh, K Matsui, S Negoro, N Yamamoto, J E Latz, S Adachi, M Fukuoka

**Affiliations:** 1 Kinki University School of Medicine, Osakasayama, 589-8511, Japan; 2 Osaka City University Medical School, Osaka, 545-8586, Japan; 3 Osaka Prefectural Medical Center for Respiratory and Allergic Diseases, Osaka, 583-8588, Japan; 4 Osaka City General Hospital, Osaka, 534-0021, Japan; 5 Shizuoka Cancer Center, Shizuoka, 411-8777, Japan; 6 Eli Lilly and Company, Indianapolis, IN, 46285, USA; 7 Eli Lilly Japan K.K., Kobe, 651-0086, Japan

**Keywords:** antifolate, lung cancer, pemetrexed, pharmacokinetics, vitamin supplementation

## Abstract

The purpose of this study was to determine the maximum tolerated dose (MTD) and recommended dose (RD) of pemetrexed with folate and vitamin B12 supplementation (FA/VB_12_) in Japanese patients with solid tumours and to investigate the safety, efficacy, and pharmacokinetics of pemetrexed. Eligible patients had incurable solid tumours by standard treatments, a performance status 0–2, and adequate organ function. Pemetrexed from 300 to 1200 mg m^−2^ was administered as a 10-min infusion on day 1 of a 21-day cycle with FA/VB_12_. Totally, 31 patients were treated. Dose-limiting toxicities were alanine aminotransferase (ALT) elevation at 700 mg m^−2^, and infection and skin rash at 1200 mg m^−2^. The MTD/RD were determined to be 1200/1000 mg m^−2^, respectively. The most common grade 3/4 toxicities were neutropenia (grade (G) 3:29, G4:3%), leucopenia (G3:13, G4:3%), lympopenia (G3:13%) and ALT elevation (G3:13%). Pemetrexed pharmacokinetics in Japanese were not overtly different from those in western patients. Partial response was achieved for 5/23 evaluable patients (four with non-small cell lung cancer (NSCLC) and one with thymoma). The MTD/RD of pemetrexed were determined to be 1200/1000 mg m^−2^, respectively, that is, a higher RD than without FA/VB_12_ (500 mg m^−2^). Pemetrexed with FA/VB_12_ showed a tolerable toxicity profile and potent antitumour activity against NSCLC in this study.

Pemetrexed (LY231514, Alimta®, Eli Lilly and Company, IN, USA) is a novel antifolate (Taylor and Patel, 1992) that is approved in the United States and a number of European Union countries , for treatment of patients with malignant pleural mesothelioma (MPM) in combination with cisplatin, and non-small cell lung cancer (NSCLC) after prior chemotherapy as a single agent. *In vitro* experiments show that pemetrexed inhibits three enzymes in folate metabolism: thymidylate synthase (TS), dihydrofolate reductase (DHFR), and glycinamide ribonucleotide formyltransferase (GARFT) (Shih *et al*, 1998). Given the schedule dependency observed preclinically, three regimens were explored in phase I studies: (1) 0.2–5.2 mg m^−2^ daily for 5 days every 3 weeks (McDonald *et al*, 1998); (2) 10–40 mg m^−2^ weekly for 4 weeks repeated every 6 weeks (Rinaldi *et al*, 1995); and (3) 50–700 mg m^−2^ every 3 weeks (Rinaldi *et al*, 1999).

The third regimen (one dose every 3 weeks) was chosen for subsequent phase II studies because of its convenient administration, ability to give repeated doses, and occurrence of objective responses. The original maximum tolerated dose (MTD) and the recommended dose (RD) was 600 mg m^−2^, but was decreased to 500 mg m^−2^ owing to toxicities experienced early in phase II studies. The initial phase I and II studies showed that myelosuppression was the principle drug-related toxicity, with a frequency of grade 3/4 neutropenia of 50% and grade 3/4 thrombocytopenia of 15% (Hanauske *et al*, 2001). Less than 10% of patients experienced gastrointestinal toxicities such as diarrhoea or mucositis. Although the prevalence of gastrointestinal toxicities and severe hematologic toxicities was low, these toxicities were associated with a high risk of mortality.

Infrequent severe myelosuppression with gastrointestinal toxicity has been observed not only for pemetrexed, but for the class of antifolates, including the DHFR inhibitor methotrexate (Morgan *et al*, 1990), the TS inhibitor raltitrexed (Maughan *et al*, 1999), and the GARFT inhibitor lometrexol (Alati *et al*, 1996; Mendelsohn *et al*, 1996). Clinical experience and nonclinical studies with methotrexate and lometrexol indicated that severe toxicity may be associated with nutritional folate status (Morgan *et al*, 1990; Alati *et al*, 1996; Mendelsohn *et al*, 1996). In fact, in the study of lometrexol, a significant effect of folate supplementation on toxicity was observed (Laohavinij *et al*, 1996). Based on these experiences, Niyikiza *et al* (2002a) investigated relationships between toxicity and baseline patient characteristics for early pemetrexed studies. They found total plasma homocysteine and methylmalonic acid levels to predict severe neutropenia and thrombocytopenia, with or without grade 3/4 diarrhoea, mucositis, or infection. Homocysteine and methylmalonic acid are known as indicators of folate and vitamin B_12_ deficiencies (Rosenberg and Fenton, 1989; Savage *et al*, 1994). Thus, it was hypothesized that a patient's risk for severe toxicity could be reduced by decreasing the levels of homocysteine and methylmalonic acid with folate and vitamin B_12_ supplementation (FA/VB_12_) (Niyikiza *et al*, 2002a).

FA/VB_12_ is now required for all patients participating in pemetrexed studies. Using this strategy, the pivotal phase III studies for MPM and NSCLC were successfully conducted with amelioration of severe drug-related toxicity (Niyikiza *et al*, 2002b; Vogelzang *et al*, 2003; Hanna *et al*, 2004).

One may expect that pemetrexed administration with supplementation would be more tolerable for patients and permit significant dose escalation above the current RD of 500 mg m^−2^. Therefore, we conducted a phase I study to determine the MTD of pemetrexed with FA/VB_12_ for Japanese patients with solid tumours and to identify the RD for subsequent Japanese phase II studies. Our secondary objectives were to investigate the safety, antitumour effect, and pharmacokinetics of pemetrexed with supplementation in Japanese patients. A similar phase I study has been conducted outside Japan, but only preliminary data are available at this time (Hammond *et al*, 2003).

## PATIENTS AND METHODS

### Patient selection

Eligible patients had histologic or cytologic diagnosis of solid cancer that was incurable by standard treatments. Patients also must have been between 20 and 75 years of age, have an Eastern Cooperative Oncology Group (ECOG) performance status of 0–2, and have an estimated life expectancy of at least 3 months. Adequate organ function was required, which included bone marrow reserve (white blood cell count 4.0–12.0 × 10^3^ mm^−3^, platelets ⩾100 × 10^3^ mm^−3^, haemoglobin ⩾9.0 g dl^−1^, and absolute granulocyte count ⩾2.0 × 10^3^ mm^−3^), hepatic function (bilirubin ⩽1.5 × upper limit of normal, aspartate/alanine transaminase (AST/ALT) ⩽2.5 × upper limit of normal, and serum albumin ⩾2.5 g dl^−1^), renal function (serum creatinine ⩽upper limit of normal and Cockcroft and Gault creatinine clearance ⩾60 ml min^−1^), and lung function (PaO2 ⩾60 torr).

Prior chemotherapy or hormone therapy was allowed if it was carried out ⩾14 days before study entry (⩾35 days for nitrosourea or mitomycin-C). Previous radiotherapy was also allowed, but only if ⩽25% of marrow was irradiated and if it was completed ⩾21 days before study entry. Pretreated patients must have recovered from all toxicities before study entry. Prior surgery was allowed if patients recovered from the effect of the operation. Patients were excluded from this study for active infection, symptomatic brain metastasis, interstitial pneumonitis, or pulmonary fibrosis diagnosed by chest X-ray, serious concomitant systemic disorders incompatible with the study, clinically significant effusions, or the inability to discontinue aspirin and other nonsteroidal anti-inflammatory agents during the study.

This study was conducted in compliance with the guidelines of good clinical practice and the Declaration of Helsinki Principles, and it was approved by the local institutional review boards. All patients gave written informed consent before study entry.

### Treatment

Pemetrexed was administered as a 10-min infusion on day 1 of a 21-day cycle. Patients remained on study unless they were discontinued because of disease progression, unacceptable adverse events, inadvertent enrollment, use of excluded concomitant therapy, cycle delay >42 days, or patient refusal.

Patients were instructed to take a daily 1 g multivitamin with 500 *μ*g of folate beginning 1 week before day 1 of cycle 1 until study discontinuation. Vitamin B_12_ (1000 *μ*g) was intramuscularly injected, starting 1 week before day 1 of cycle 1 and repeated every 9 weeks until study discontinuation.

Patients enrolled in pemetrexed clinical studies have received dexamethasone prophylactically to avoid pemetrexed-induced rash. As this was the first study of pemetrexed in Japanese patients and the incidence of the drug-induced rash in Japanese patients was unknown, the steroid was not to be administered prophylactically.

### Dose escalation

In this study, 10 dose levels of pemetrexed, 300, 500, 600, 700, 800, 900, 1000, 1200, 1450, and 1750 mg m^−2^, were to be examined with a starting dose of 300 mg m^−2^. At dose levels from 300 to 1000 mg m^−2^, three patients were to be treated initially. If no dose-limiting toxicities (DLTs) occurred during cycle 1, escalation proceeded to the next dose level. If 1 DLT occurred, three patients were added. If no additional DLTs were observed, escalation proceeded to the next dose level. At dose levels from 1200 to 1750 mg m^−2^, six patients were to be treated at once. If two or more patients had DLTs at any dose level, dose escalation stopped, and this dose level was considered the MTD. The RD was then established by discussion with principal investigators, and the Efficacy and Safety Evaluation Committee.

A DLT was defined as the occurrence of one of the following toxicities during cycle 1: any grade 3/4 nonhematologic toxicity (except grade 3 nausea/vomiting and AST, ALT, or alkaline phosphatase elevation <10 × upper limit of normal that returns to grade 0–1 by the beginning of cycle 2), grade 3/4 febrile neutropenia (<1000 mm^−3^ with ⩾38.0°C), grade 4 leucopenia (<1000 mm^−3^) or neutropenia (<500 mm^−3^) lasting ⩾4 days, thrombocytopenia (<20 000 mm^−3^), or thrombocytopenia (⩾20 000 mm^−3^) requiring platelet transfusion. A failure to start the second cycle by day 42 owing to toxicity was also considered a DLT. All toxicities were assessed according to National Cancer Institute-Common Toxicity Criteria (NCI-CTC) version 2.

### Treatment assessments

Tumour response was assessed by the Response Evaluation Criteria in Solid Tumors (RECIST) criteria. Evaluable patients were subjected to CT or MRI measurement to determine the size of tumours at anytime at the discretion of investigators.

### Pharmacokinetic analysis

Blood and urine were collected from each patient over a period of 72 h following administration in cycle 1. Blood samples were taken just before administration, at the end of infusion, and approximately 5, 15, 30 min and 1, 2, 4, 6, 8, 24, 48 and 72 h after the start of infusion. Urine was collected over the following time intervals: 0–4, 4–8, 8–12, 12–24, 24–36, 36–48, 48–60, and 60–72 h. Plasma and urine samples were analysed for pemetrexed at Taylor Technology Inc., Princeton, NJ, USA. Plasma samples were analysed using a validated liquid chromatography/electrospray ionisation-tandem mass spectrometry method that generated a linear response over the concentration ranges of 10–2000 ng/ml and 1000–200 000 ng/ml (Latz *et al*, 2006). Urine samples were analysed using a similar analytical technique (Chaudhary *et al*, 1999).

Pharmacokinetics were evaluated using noncompartmental methods (WinNonlin Professional Version 3.1; Pharsight Corporation, Cary NC, USA). Pharmacokinetic parameters determined based on plasma concentration *vs* time data were maximum plasma concentration (*C*_max_), elimination half-life (*t*_1/2_), area under the plasma concentration *vs* time curve (AUC) from time 0 to infinity (AUC_0-∞_), volume of distribution at steady-state (*V*_ss_) and plasma clearance (CL_p_) (Rowland and Tozer, 1995). The fraction of drug excreted unchanged in urine (*F*_e_) was calculated by dividing the cumulative amount of pemetrexed excreted unchanged in urine within 72 h (Ae_0–72_) by the administered dose (Rowland and Tozer, 1995).

## RESULTS

### Patient disposition and characteristics

From October 2001 to September 2004, a total of 35 Japanese patients were enrolled and 31 were treated at four centres in Japan. Four patients were not treated owing to protocol criteria not met (*n*=3) and investigator decision (*n*=1). The majority of patients were male (65%), had an ECOG performance status of 1 (84%), were diagnosed with NSCLC (61%), and received prior chemotherapy (94%) (Table 1).

### Dose escalation and dose-limiting toxicities

Three or six patients were enrolled at each dose level from 300 to 1200 mg m^−2^, except the 900 mg m^−2^ dose level (Table 2). At this dose level, one additional patient was enrolled because a patient was excluded from the DLT analysis. Before the dose initiation, this patient had grade 3 fasting hyperglycemia that was aggravated after the start of dosing. Therefore, this patient was rated as inappropriate for evaluation.

The first DLT was observed at the 700 mg m^−2^ dose level. This 66-year-old woman with NSCLC experienced grade 3 ALT elevation. After an additional three patients were enrolled, no other DLTs were observed.

The next DLTs were observed at the 1200 mg m^−2^ dose level, which enrolled six patients at once. One patient, a 72-year-old woman with MPM, had grade 3 infection at day 6 of cycle 1. Neutropenia was not simultaneously observed in this cycle. After 12 days, the event was resolved with antibiotics. This patient continued in study with dose reduction to 1000 mg m^−2^. The other patient, a 68-year-old man with NSCLC, had grade 2 rash at day 5 of cycle 1. The severity of the event reached grade 3 at day 7. After 9 days from the occurrence, rash was resolved with dexamethasone and H_1_-antihistamine. This patient continued in study without dose reduction. As two DLTs were observed, the 1200 mg m^−2^ dose level was considered as the MTD. The RD for subsequent phase II studies was then evaluated to be pemetrexed 1000 mg m^−2^. Both events were considered as drug-related events by investigators.

### Safety

The safety evaluation was completed from data obtained from cycle 1–6 for all dose levels except 1200 mg m^−2^ (cycle 1–3). These data were collected and analysed to evaluate safety when the MTD and RD were determined. The major toxicities observed in >50% of patients during all cycles evaluated for this report included rash, nausea, anorexia, fatigue, ALT elevation, AST elevation, lactate dehydrogenase elevation, leucopenia, neutropenia, lymphopenia, hematocrit decreased, haemoglobin decreased and erythropenia (Table 3). The most commonly reported grade 3/4 toxicity was neutropenia: nine patients (29%) had grade 3 neutropenia, and one patient (3%) had grade 4 neutropenia. Other grade 3/4 hematologic toxicities were grade 3 leucopenia in four patients (13%), grade 4 leucopenia in one patient (3%), grade 3 lymphopenia in four patients (13%), and grade 3 haemoglobin decreased in two patients (6%). The most commonly reported grade 3 nonhematologic toxicity was ALT elevation (four patients (13%)). Other grade 3 toxicities included AST elevation in one patient (3%), anorexia in one patient (3%), infection in one patient (3%), malaise in one patient (3%), and rash in one patient (3%) were observed. No grade 4 nonhematologic toxicities were reported.

The only serious adverse event was observed at the 900 mg m^−2^ level. This 71-year-old man with NSCLC experienced grade 1 pyrexia at day 18 of cycle 3 and was hospitalized; however, the event was resolved the next day. The investigator did not consider it as a drug-related event. One patient at 900 mg m^−2^ level discontinued treatment owing to adverse events (neutropenia, anorexia, and pyrexia). No deaths were observed during the study period or for 31 days after the last dose.

At the 900 mg m^−2^ and higher dose levels, all patients had either grade 1/2 or grade 3/4 rash. At cycle 1, 25 patients experienced rash. Of these, 20 patients received corticosteroid. At or after cycle 2, corticosteroid treatment was given only for nine rash events, whereas rash events were observed in 20 cycles in cumulative total among patients. In addition, the severity of rash quickly improved or disappeared after administration of corticosteroid. Although the protocol allowed corticosteroid use for prevention of rash from cycle 2, only seven patients actually received the preventive treatment. Among those who did not receive the prophylactic corticosteroid, the incidence of a rash observed at, or after, cycle 2 was about one-third of the incidence observed in cycle 1.

### Pharmacokinetic analysis

**Figure 1:**
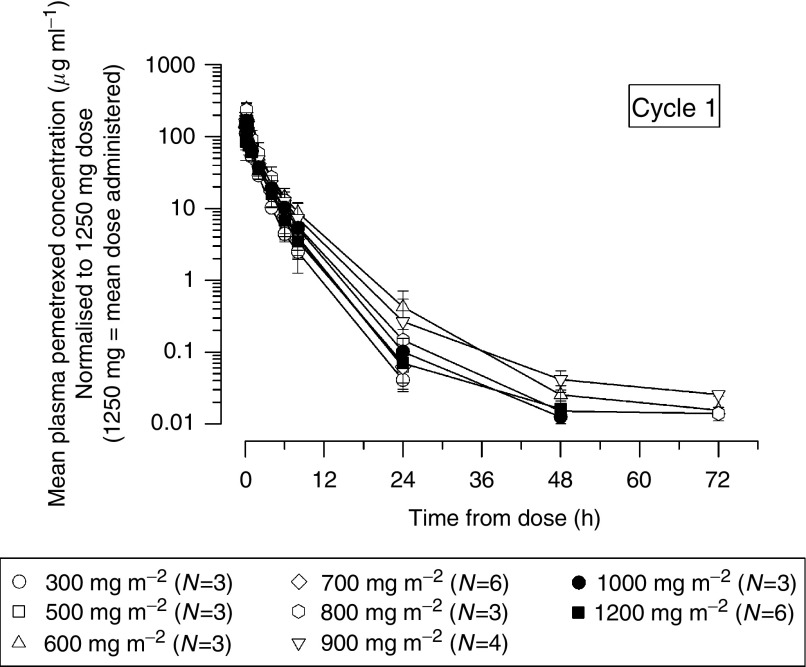
Mean dose-normalised pemetrexed plasma concentration–time profiles following single-dose administration in Japanese patients.

Mean dose-normalised pemetrexed plasma concentration *vs* time profiles following single doses of 300–1200 mg m^−2^ pemetrexed are provided in Figure 1. This body surface area (BSA)-normalized dose range represents absolute doses of 414–2018 mg in Japanese patients with a mean BSA of 1.64 m^2^ (range, 1.36–1.97 m^2^).

Pharmacokinetic parameters for each dose group are summarised in Table 4. Lack of a monotonic trend in CL_p_ and V_ss_ between cohorts indicated that pemetrexed pharmacokinetics are consistent across dose groups. Consistency of pemetrexed pharmacokinetics across dose groups is also illustrated by the lack of systematic pattern across dose groups in the dose-normalised plasma concentration *vs* time profiles (Figure 1). The overall mean *t*_1/2_ is approximately 2.74 h and was essentially similar across all dose groups (range, 2.28–3.62 h).

In this study, pemetrexed was primarily excreted unchanged in urine, which is consistent with its known elimination pathway (i.e., renal excretion). The *F*_e_ averaged 0.752 (range, 0.645–0.827). Mean *F*_e_ values were consistent across dosing cohorts.

### Tumour response

In this study, 23 of the 31 patients were evaluable for response by RECIST criteria (Table 5). Partial responses (PRs) were observed in four patients with NSCLC (one patient each at 500, 700, 800, and 1200 mg m^−2^) and one patient with thymoma at 500 mg m^−2^. In addition, one patient with NSCLC at 500 mg m^−2^ had a PR by the World Health Organization criteria, but was not evaluable via RECIST.

## DISCUSSION

This is the first phase I study of pemetrexed in Japanese patients. The MTD for pemetrexed administered with FA/VB_12_ was 1200 mg m^−2^ and determined the RD for subsequent phase II studies was 1000 mg m^−2^.

In contrast with the previously determined MTD (600 mg m^−2^) without vitamin supplementation (Rinaldi *et al*, 1999), our MTD increased by a factor of 2 whereas maintaining a tolerable safety profile. Niyikiza *et al* (2002a, 2002b) conducted a multivariate analysis on 246 patients in phase II pemetrexed studies without vitamin supplementation, and the incidence of grade 4 neutropenia was 32% and grade 4 thrombocytopenia was 8% . Also 6% of patients had grade 3/4 diarrhoea, 5% had grade 3/4 mucositis, and a 5% incidence of drug-related death occurred. In contrast, our study had grade 4 neutropenia of only 3% (one patient) and no grade 4 thrombocytopenia. In addition, no grade 3/4 diarrhoea or mucositis, and no drug-related deaths were observed.

In the pivotal phase III study of NSCLC patients, those who received pemetrexed (500 mg m^−2^) plus vitamin supplementation had a lower incidence of severe toxicities compared to those who received docetaxel (75 mg m^−2^), including grade 3/4 neutropenia (5.3 *vs* 40.2%) and grade 3/4 diarrhoea (0.4 *vs* 2.5%) (Hanna *et al*, 2004).

Dose-dependency for toxicity of pemetrexed plus supplementation was further investigated to understand the effect of supplementation on safety. The patients in this study were divided into three groups by doses: low dose (300–600 mg m^−2^ (*n*=9)), middle dose (700–900 mg m^−2^ (*n*=13)), and high dose (1000 and 1200 mg m^−2^ (*n*=9)). Grade 1/2 toxicity such as erythropenia, lymphopenia, hematocrit decreased, ALT and AST elevation, and anorexia increased dose dependently from approximately 20–50% to approximately 75%. However, there was no obvious correlation between grade 3/4 toxicity and dose group. Therefore, high dose levels of pemetrexed with FA/VB_12_ is expected to be tolerable enough for patients.

In this study, severe rash was rarely observed even without the prophylactic corticosteroid. Although this result suggests that the steroid premedication for prevention of severe rash is no longer necessary for patients with pemetrexed treatment if the FA/VB_12_ is concomitantly conducted, it would be too early to conclude it as the data of patients untreated with the premedication are limited at this moment.

The pharmacokinetic results in our study were consistent with a phase I study of pemetrexed without vitamin supplementation in western patients by Rinaldi *et al* (1999) In that study, pemetrexed *t*_1/2_ was 3.1 h; and CL was 85 ml/min (Rinaldi *et al*, 1999 and unpublished results). In our study, the *t*_1/2_ of pemetrexed was about 2.7 h; and CL was 81.9 ml/min. Additionally, the *F*_e_ of pemetrexed was similar for Japanese patients (75% in our study) and western patients (78% in the Rinaldi study (Rinaldi *et al*, 1999)). These results indicate that pharmacokinetics of pemetrexed in Japanese patients are similar to those in western patients.

Although our study is the first phase I study to evaluate pemetrexed with FA/VB_12_ in Japanese patients, a similar phase I study has been conducted in western patients. In the preliminary results of that study, heavily pretreated patients had a MTD of 925 mg m^−2^, and lightly pretreated patients had a MTD of 1050 mg m^−2^ (Hammond *et al*, 2003). The comparison of these two studies suggests that the improved tolerability experienced by Japanese patients when pemetrexed is administered with FA/VB_12_ is not attributable to ethnic differences; rather, it is attributable to the vitamin supplementation.

In our phase I study, four NSCLC patients and one thymoma patient had PRs. Except for one, all of the patients with PR had ⩾3 prior chemotherapy regimens. The NSCLC patients with PRs received doses of pemetrexed higher than 500 mg m^−2^, which is the approved dose for NSCLC treatment in a number of countries. Therefore, subsequent phase II studies using our RD of 1000 mg m^−2^ with vitamin supplementation could show more prominent antitumour activity for cancer patients. To examine this hypothesis, a Japanese phase II study is being conducted, examining pemetrexed 500 or 1000 mg m^−2^ every 3 weeks with full supplementation for patients with locally advanced or metastatic NSCLC. Clinical trials for other tumours, including MPM, are also ongoing. For the prophylactic corticosteroid, as severe rash was not frequently observed in this study, the steroid is not to be administered prophylactically in both currently on-going studies.

In conclusion, pemetrexed with FA/VB_12_ resulted in a tolerable toxicity profile. The MTD was 1200 mg m^−2^. The RD was 1000 mg m^−2^.

## Figures and Tables

**Table 1:** Baseline patient characteristics

**Parameter**	***N*=31**
*Sex, n* (%)	
Male	20 (65)
Female	11 (35)
	
*Age, years*	
Median (range)	59 (31–74)
Mean (s.d.)	57 (11)
	
*ECOG performance status, n* (%)	
0	4 (13)
1	26 (84)
2	1 (3)
	
*Diagnosis, n* (%)	
Non-small cell lung cancer	19 (61)
Malignant pleural mesothelioma	7 (23)
Thymoma	2 (7)
Alveolar soft part sarcoma	1 (3)
Rectal cancer	1 (3)
Unknown primary cancer	1 (3)
	
*Prior therapy, n* (%)	
Surgery	14 (45)
Radiation	9 (29)
Chemotherapy	29 (94)

ECOG=Eastern Cooperative Oncology Group; s.d.=standard deviation.

**Table 2:** Dose escalation and DLTs

**Dose (mg m^−2^)**	**Number of patients**	**DLTs (*n*)**
300	3	None
500	3	None
600	3	None
700	6	G3 ALT elevation (1)
800	3	None
900	4^a^	None
1000	3	None
1200	6	G3 infection (1); G3 rash (1)

ALT=alanine transaminase; DLT=dose-limiting toxicity; G3=grade 3.

a One patient was excluded for DLT analysis because of grade 3 hyperglycemia at the beginning of the study.

**Table 3:** Incidence of clinically relevant toxicities

	**Dose (mg m^−2^) (*n*)**															
	**Grade**															
	**300 (3)**		**500 (3)**		**600 (3)**		**700 (6)**		**800 (3)**		**900 (4)**		**1000 (3)**		**1200 (6)**	
**Toxicity**	**1/2**	**3/4**	**1/2**	**3/4**	**1/2**	**3/4**	**1/2**	**3/4**	**1/2**	**3/4**	**1/2**	**3/4**	**1/2**	**3/4**	**1/2**	**3/4**
*Hematologic*																
Erythropenia	1	0	1	0	3	0	4	0	2	0	2	0	2	0	5	0
Hematocrit decreased	1	0	1	0	3	0	4	0	3	0	2	0	2	0	5	0
Haemoglobin decreased	2	0	2	0	2	0	3	0	2	0	1	1	2	0	4	1
Leucopenia	1	0	3	0	2	1	3	1	1	1	1	1	1	0	5	1
Lymphopenia	0	0	2	1	0	1	3	0	1	0	1	1	3	0	4	1
Neutropenia	1	0	1	2	1	2	3	2	0	2	1	1	2	0	2	1
Thrombocytopenia	0	0	2	0	1	0	2	0	2	0	2	0	1	0	2	0
																
*Nonhematologic*																
ALT elevation	0	0	2	0	2	0	2	3	3	0	1	1	1	0	5	0
AST elevation	0	0	3	0	2	0	4	1	3	0	3	0	2	0	5	0
Blood bilirubin increased	0	0	1	0	0	0	2	0	0	0	0	0	0	0	1	0
LDH elevation	0	0	3	0	3	0	5	0	3	0	2	0	1	0	4	0
Alopecia	0	0	0	0	2	0	2	0	1	0	2	0	0	0	0	0
Anorexia	0	0	1	0	3	0	5	0	3	0	0	1	3	0	4	0
Constipation	1	0	1	0	0	0	1	0	0	0	0	0	2	0	1	0
Diarrhoea	0	0	2	0	1	0	1	0	1	0	1	0	1	0	2	0
Fatigue	1	0	2	0	2	0	2	0	3	0	1	0	2	0	3	0
Infection	0	0	0	0	0	0	2	0	0	0	0	0	0	0	0	1
Nausea	2	0	3	0	3	0	5	0	3	0	2	0	2	0	5	0
Malaise	0	0	0	0	1	0	0	1	0	0	0	0	0	0	0	0
Pruritus	0	0	0	0	2	0	2	0	1	0	0	0	1	0	2	0
Rash	3	0	2	0	3	0	5	0	2	0	4	0	3	0	5	1
Vomiting	2	0	3	0	2	0	3	0	1	0	1	0	1	0	0	0

ALT=alanine transaminase; AST=aspartate transaminase; LDH=lactate dehydrogenase.

**Table 4:** Summary of pemetrexed pharmacokinetic parameters by dosing cohort arithmetic mean (CV%)

	**Dose (mg m^−2^) (*n*)**							
**Parameter**	**300 (3)**	**500 (3)**	**600 (3)**	**700 (6)**	**800 (3)**	**900 (4)**	**1000 (3)**	**1200 (6)**
Dose (mg)	459 (12.4%)	783 (7.56%)	919 (8.28%)	1180 (8.06%)	1280 (16.5%)	1550 (5.47%)	1820 (7.04%)	1910 (6.71%)
*C*_max_, (*μ*g ml^−1^)	58.2 (7.15%)	115 (19.1%)	178 (15.7%)	172 (9.30%)	240 (14.5%)	217 (7.05%)	269 (17.8%)	212 (13.2%)
AUC_0-∞,_ (*μ*g h ml^−1^)	70.1 (7.04%)	158 (21.6%)	290 (12.5%)	250 (23.5%)	361 (17.0%)	388 (19.6%)	382 (6.55%)	337 (24.6%)
CL_p_ (ml min^−1^)	109 (5.89%)	86.5 (32.5%)	53.0 (3.95%)	83.4 (27.7%)	61.4 (35.2%)	68.5 (20.0%)	79.3 (2.57%)	99.7 (24.7%)
*V*_ss_ (l)	13.5 (22.2%)	12.1 (20.1%)	11.5 (25.5%)	11.7 (20.0%)	10.6 (33.6%)	13.9 (31.7%)	14.4 (7.40%)	14.8 (9.41%)
*t*_1/2_ (h)	2.28 (25.2%)	2.62 (3.29%)	3.62 (28.7%)	2.51 (3.91%)	2.93 (14.6%)	3.02 (17.8%)	2.67 (1.90%)	2.55 (10.9%)
*F*_e_	0.659 (8.78%)	0.645 (8.34%)	0.788 (3.76%)	0.807 (10.1%)	0.705 (34.9%)	0.797^a^ (5.11%)	0.648^a^ (12.5%)	0.827^a^ (7.58%)

CV%=coefficient of variation expressed as a percentage; *C*_max_=maximum observed drug concentration; AUC_0-∞_=area under the concentration versus time curve from zero to infinity; CL=total body clearance of drug after intravenous administration; *V*_ss_=volume of distribution at steady state; *t*_1/2_=half-life associated with the terminal rate constant; *F*_e_=fraction of dose eliminated unchanged in urine.

a The numbers of patients in 900, 1000, and 1200 mgm^−2^ were three, two, and five, respectively, owing to incompletion of urine collections for patients 209, 210, and 306.

**Table 5:** Antitumour activity by dose (RECIST)

		**Evaluable (*n*=23)**				
**Dose** **(mg m^−2^)**	**Number of patients**	**CR**	**PR^a^**	**s.d.**	**PD**	**NE**
300	3	0	0	2	0	1
500	3	0	2	0	0	0
600	3	0	0	1	0	0
700	6	0	1	3	1	0
800	3	0	1	0	1	1
900	4	0	0	2	0	1
1000	3	0	0	1	1	0
1200	6	0	1	2	1	0
Total	31	0	5	11	4	3

NSCLC=non-small cell lung cancer; CR=complete response; NE=not evaluated; PD=progressive disease; PR=partial response; s.d.=stable disease.

a In addition, one NSCLC patient at 500 mg m^−2^ had PR via WHO criteria.
